# The Interplay Between Epigenetic Regulation and CD8^+^ T Cell Differentiation/Exhaustion for T Cell Immunotherapy

**DOI:** 10.3389/fcell.2021.783227

**Published:** 2022-01-11

**Authors:** Wai Ki Wong, Bohan Yin, Ching Ying Katherine Lam, Yingying Huang, Jiaxiang Yan, Zhiwu Tan, Siu Hong Dexter Wong

**Affiliations:** ^1^ Department of Bioengineering, Imperial College London, London, United Kingdom; ^2^ Department of Biomedical Engineering, The Hong Kong Polytechnic University, Kowloon, Hong Kong, China; ^3^ AIDS Institute and Department of Microbiology, The University of Hong Kong, Pokfulam, Hong Kong, China

**Keywords:** T-cell differentiation, T-cell exhaustion, epigenetic regulation, adoptive immunotherapy, T-cell activation

## Abstract

Effective immunotherapy treats cancers by eradicating tumourigenic cells by activated tumour antigen-specific and bystander CD8^+^ T-cells. However, T-cells can gradually lose cytotoxicity in the tumour microenvironment, known as exhaustion. Recently, DNA methylation, histone modification, and chromatin architecture have provided novel insights into epigenetic regulations of T-cell differentiation/exhaustion, thereby controlling the translational potential of the T-cells. Thus, developing strategies to govern epigenetic switches of T-cells dynamically is critical to maintaining the effector function of antigen-specific T-cells. In this mini-review, we 1) describe the correlation between epigenetic states and T cell phenotypes; 2) discuss the enzymatic factors and intracellular/extracellular microRNA imprinting T-cell epigenomes that drive T-cell exhaustion; 3) highlight recent advances in epigenetic interventions to rescue CD8^+^ T-cell functions from exhaustion. Finally, we express our perspective that regulating the interplay between epigenetic changes and transcriptional programs provides translational implications of current immunotherapy for cancer treatments.

## Introduction

Adaptive immunity is a physiological defensive mechanism, including fighting against cancers. T cell-based adaptive immunity is one of the current immunotherapies that relies on the recognition and lysis of target cancer cells to achieve an effective anti-tumor response (Wong W. K. et al., 2021). In the tumor microenvironment (TME), dendritic cells (DCs) phagocytose apoptotic tumorigenic cell antigens and present the antigen as peptide-major histocompatibility complex (pMHC). The DCs subsequently migrate into the lymph node to prime naïve T cells through engaging the pMHC with T cell receptor (TCR) (Albert et al., 1998; Mempel et al., 2004; Bottcher and Sousa, 2018; Hiam-Galvez et al., 2021). However, TCR is a highly specific biomolecular construct that can vary T cell responses to cognate antigen with minor changes in the peptide sequence of the TCR (Liu Y. et al., 2016). Subsequently, the activated T cells with antigen-specific TCR from a T cell pool in lymph nodes undergo differentiation, migrate and infiltrate into TME to attack target cancer cells (Bousso, 2008; Gattinoni et al., 2012; Ozga et al., 2016; van der Woude et al., 2017). We mainly focus on the physiology of CD8^+^ T cells against chronic antigen exposure or cancer in this review.

**FIGURE 1:**
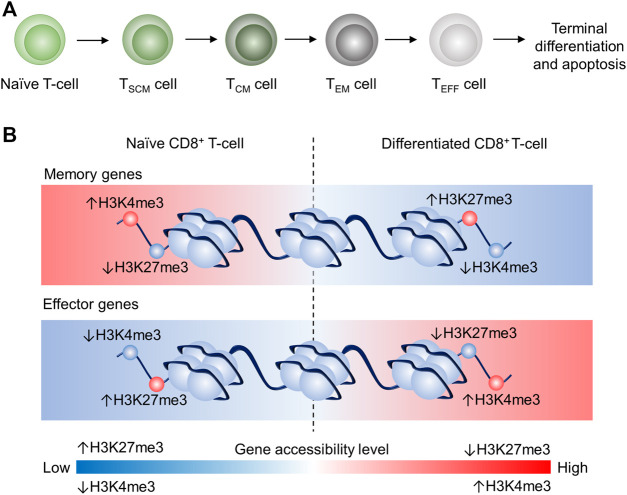
Schematic illustration of the proposed linear model and epigenetic remodeling through dynamic histone modification of CD8^+^ T cell differentiation CD8^+^ T cell differentiation (Crompton et al., 2016; Henning et al., 2018b). **(A)** Progressive acquisition of effector function by naïve CD8^+^ T-cell differentiation: Naïve (T_N_)→T_SCM_→T_CM_→T_EM_→T_EFF_, which will eventually terminate differentiation and undergo apoptosis. **(B)** Schematic illustrating of the epigenetic remodeling dynamics of H3K4me3 and H3K24me3 in T cells. In T_N_, memory genes are more accessible because of high H3K4me3 tag density and low H3K27me3 tag density, whereas effector genes in T_N_ are less accessible due to low H3K4me3 tag density and high H3K27me3 tag density. In differentiated CD8^+^ T cells, memory genes are less accessible due to low H3K4me3 tag density and high H3K27me3 tag density, whereas effector genes in CD8^+^ differentiated T cells are more accessible because of high H3K4me3 tag density and low H3K27me3 tag density. This epigenetic remodeling is associated with the memory gene downregulation and effector gene upregulation in differentiated CD8^+^ T cells.

Activated T cells differentiate into T cell subsets with various biological functions in adaptive immunity. Previous studies proposed a progressive T cell differentiation model to describe the T cell lineage relationship depending on signal strength (Henning et al., 2018a). Gattinoni et al. suggested that naïve T cell (T_N_) differentiated in the following order: memory stem T (T_SCM_) cell, central memory T (T_CM_) cell, effector memory T (T_EM_), effector T (T_E_) cell as illustrated in Figure 1A (Huster et al., 2006; Gattinoni et al., 2012; Henning et al., 2018a). Each subset has its feature in terms of lifespan and T cell activity. For instance, T_SCM_ is a recently identified subset that possesses stem cell characteristics: self-renewal and the potency of differentiation into T_CM_, T_EM_, T_E_. Also, T_SCM_ showed the highest proliferation capacity among T cell subsets (including T_N_) and higher anti-tumor activity than T_CM_, T_EM_, and T_E_ (Gattinoni et al., 2011). Memory T cells can respond to antigen challenges with robust expansion *in vivo* and show cytolytic features earlier than T_N_ cells (Croft, 1994; Bruno et al., 1995; Cho et al., 1999; Veiga-Fernandes et al., 2000; Berard and Tough, 2002; Joshi and Kaech, 2008). T_CM_ and T_EM_ are two longer living subsets than T_E_. T_CM_ is defined as CC-chemokine receptor 7 positive (CCR7^+^) and able to return into secondary lymphoid organs (e.g., lymph node and spleen (Ruddle and Akirav, 2009)), while T_EM_ is defined as CCR7^–^ and can accumulate in inflamed tissues and shows more prominent effector feature, including higher production of interferon-gamma (IFNγ), compared to T_CM_ (Sallusto et al., 1999; Sallusto et al., 2004; Huster et al., 2006). T_E_ is short-lived and the terminal phenotype of T cells in the differentiation lineage, according to the proposed model. T_E_ is featured by more effector molecules storage such as granzyme B (GZMB) in granules and higher early cytotoxicity (<6 h) *in vitro* compared to T_CM_, T_EM_ cells (Wolint et al., 2004; Huster et al., 2006).

However, human adaptive immunity often fails to clear tumors completely, leading to the progressive growth of cancer cells and metastasis without medical intervention. One key challenge to this self-defense is T cell exhaustion, which promotes apoptosis and the upregulation of inhibitory receptors as a state of T cell dysfunction (Wherry, 2011). Inhibitory ligands, such as programmed death-ligand 1 (PD-L1) expressed on cancer cells, bind to the corresponding inhibitory receptor, programmed cell death protein 1 (PD-1), expressed on T cells, leading to inhibition of T cell activation signals (Boussiotis, 2016; Wong S. H. D. et al., 2021). Thus, T cell exhaustion weakens adaptive immunity that causes the failure of self-defense against tumors. To address this issue, researchers have developed immune-checkpoint blockade strategies to disrupt the interaction between inhibitory ligands and receptors, thereby preventing inhibitory signals in T cells (Leach et al., 1996; Freeman et al., 2000; Iwai et al., 2005). This strategy has shown improved therapeutic outcomes for cancers (Hamid et al., 2013; Robert et al., 2014; Tumeh et al., 2014; Ansell et al., 2015).

Therefore, versatile biological signals can regulate CD8^+^ T cell behaviors. Stimulatory/inhibitory signals initiate intracellular signal cascades through T-cell membrane receptors and alter gene expression profiles that ultimately define T cell functionality and phenotype (Gattinoni et al., 2012; Chen and Flies, 2013; Boussiotis, 2016). More importantly, those signals indirectly modulate gene methylation and histone modification (methylation of histone protein tail). Hence, T cell differentiation and T cell exhaustion are two critical determinants in anti-tumor activities by adaptive immunity. It is highly desirable to explore the mechanistic insights into epigenetic regulation of T cell behaviors for engineering T cell-based immunotherapy strategies to treat cancers (Hiam-Galvez et al., 2021). Several recent reviews have explored the microRNA (miRNA) involved in the regulations of T cell differentiation and malignancy (Liang et al., 2015; Saki et al., 2015; Rodríguez-Galán et al., 2018). However, limited reviews discuss the association between miRNA and the enzymatic factors to govern epigenetics of CD8^+^ T cells against tumors and pathogens. In this mini-review, we discuss the correlated epigenetic profile of the respective CD8^+^ T cell phenotype, highlight the significant enzymatic factors and intracellular/extracellular miRNA that regulate T cell response at the epigenetic level, and discuss the possible strategies by manipulating these factors for improved cancer treatments.

## Epigenetic Landscape Correlates With T Cell Differentiation

The epigenetic landscape represents the gene accessibility profile, which describes the physical occupancy of chromatin-binding molecules (such as transcription factor and polymerase) along with the sequences of interest (Klemm et al., 2019), typically revealed by assay for transposase-accessible chromatin with sequencing (ATAC-Seq). Such assay describes the transcription availability of the chromatin physical openness. Avgustinova et al. reported that the controlled and dynamic epigenetic landscape underlined the stem cell fate of both haematopoietic and mesenchymal lineages (Avgustinova and Benitah, 2016), responsible for replacing damaged or dying cells during tissue homeostasis. In the immune system, CD8^+^ T cells play a central role in defending against pathogen infection (Harty et al., 2000) and tumors (Yee et al., 2002). The global epigenetic difference lies in the gene loci associated with activated T cell features such as cell division, immune response, and metabolism (Scharer et al., 2017). Specifically, CD8^+^ T_N_, T_E_, and memory T cells showed distinct genome-wide accessibility, suggesting the epigenetic remodeling during T cell differentiation. Scharer et al. showed that the global chromatin accessibility of lymphocytic choriomeningitis virus (LCMV) infection-activated CD8^+^ T_N_ was dynamically remodeled at the indicated time points (Scharer et al., 2017). Critically, their results indicated that T_N_ progressively/linear changed to memory T cells in terms of chromatin accessibility and most memory-related differentially accessible regions shared with effector cells, with one exceptional small subset that was reprogrammed during differentiation from effector memory. These findings potentially compel the construction of the recently established linear/circular model during T cell differentiation (Henning et al., 2018b). These global views on epigenome indicate the importance of understanding epigenetic mechanisms underlying T cell differentiation and related immune response. These reports motivate us to discuss the effect of individual epigenetic modifications, such as H3K27me3 (trimethylation of lysine 27 on histone H3) and H3K4me3 modifications as the recently investigated candidates, on CD8^+^ T cell differentiation in this mini-review.

DNA methylation silences gene expressions by restricting accessibility to transcription machinery, including the recruitment of transcription factors and RNA polymerase II, through methylating cytosines without interfering with DNA sequences (epigenetics) (Keshet et al., 1986; Cedar, 1988; Moore et al., 2013; Davies et al., 2014; Yang et al., 2014). Thus, DNA methylation (“OFF”) and demethylation (“ON”) regulate gene expression profiles, thereby predicting T cell phenotypes upon cell differentiation (Cedar, 1988; Robertson, 2005; Moore et al., 2013; Smith and Meissner, 2013). To verify the proposed linear T cell differentiation model, recent research has investigated progressive epigenetic changes across various T cell subtypes from T_N_. For instance, Yang et al. has recently revealed a methylome analysis on T cells isolated from colorectal cancer patients. The authors showed that DNA methylation levels of signature genes are associated with T cell phenotypes: *Tcf7*, a naïve characteristic gene, was demethylated in T_N_, but highly methylated in T_EM_ cells and tumor-infiltrating lymphocytes (TILs); *Ifng* and *Gzmb*, known as cytotoxic characteristic genes, were highly methylated in T_N_, but demethylated in T_EM_ cells (Yang et al., 2019). The “opposing” methylation state features of both T_N_ signature genes and cytotoxic signature genes might facilitate the T cell differentiation. Besides, it is possible to probe T cell subsets by recognizing the respective methylome profile.

Histone modification regulates gene transcription by changing the gene accessibility, including the modulation of physical chromatin compaction (Lawrence et al., 2016). Thus, histone modification enriched at T cell function-associated genes significantly influence gene expression. Acetylation (ac) of histone (e.g., H3 lysine 9/H3K9) is associated with accessible chromatin structure for transcription (Fann et al., 2006). An early report from Fann et al. showed that a higher level of H3K9ac was detected in several cytokine loci (such as *IL2* and *TNF*) in resting memory CD8^+^ T cells compared to resting T_N_ cells (Fann et al., 2006). Upon stimulation, the transcription activity of these genes was higher in activated memory CD8^+^ T cells compared to activated T_N_ cells (Fann et al., 2006). These findings suggest that histone modification could control T cell response to stimulation through transcription regulation, and differences in the epigenetic landscape might define the functional difference of distinct T cell subsets.

H3K4me3 (Histone 3 Lysine 4 trimethylation) and H3K27me3 (Histone 3 Lysine 27 trimethylation) are believed in the regulation of stem cell differentiation (Margueron and Reinberg, 2011). H3K4me3 and H3K27me3 are also two well-known examples of CD8^+^ T cell differentiation (Crompton et al., 2016). Specifically, memory genes (e.g., *Tcf7)* are downregulated during CD8^+^ T with reduced H3K4me3 tag and increased H3K27me3 tag at memory gene promoter regions (Figure 1B). However, effector genes (e.g., *Tbx21*) are upregulated in differentiated CD8^+^ T cells (e.g., T_CM_ and T_EM_) with increased H3K4me3 tag and reduced H3K27me3 tag at effector gene promoter regions (Crompton et al., 2016). Therefore, dynamics of methylome profile and histone methylation correlate to the lineage along with the linear model.

T-cell development involves roles for some transcription factors that are products of multigene families, such as the ETS family and the RUNX family that are likely to play important roles as participants in most lymphoid gene expression, based on the extreme enrichment of their binding motifs in enhancers of lymphoid genes with various patterns of activity (Rothenberg et al., 2016). In particular, Russell et al. showed that ETS-1 regulated cytokine production in T cells, and ETS-1 deficient CD8^+^ T cells significantly reduced the expression of IL-2 and IFNγ (Russell and Garrett-Sinha, 2010). Besides, ETS-1 was shown to be essential for T cell survival (Bories et al., 1995; Muthusamy et al., 1995). In the context of CD8^+^ T cell differentiation, Grennigingloh et al. revealed that ETS-1 maintained the expression of IL-7 receptor responsible for memory T cell development (Grenningloh et al., 2011). Besides, ETS-1 is also shown to regulate the expression of the IL-12 receptor, responsible for effector T cell development (Li et al., 2010; Liu M. et al., 2016). Therefore, ETS-1 is likely to be an essential transcription factor for facilitating CD8^+^ T cell differentiation. However, the report of the relation between ETS-1 and overall CD8^+^ T cell-dependent anti-tumor response *in vivo* has been rare at this stage.

ETS-1 also mediates the expression of Runt-related transcription factor 3 (Runx3) (Zamisch et al., 2009; Liu M. et al., 2016), a well-known transcription factor involved in CD8 lineage commitment from thymic double-positive (CD4^+^CD8^+^) T cells (Sato et al., 2005) and T_E_ cell development (Shan et al., 2017). Recently, Milner and colleagues showed that Runx3 was responsible for generating tissue-resident memory T (T_RM_) cells (Milner et al., 2017). Interestingly, the authors showed that activated T cells showed increased *tbx21* (encoding T-Bet, an immune cell-specific member of the T-box family of transcription factors) accessibility to Runx3 compared to T_N_ cells. The authors also indicated that direct binding of Runx3 on *tbx21* loci inhibited the expression of T-bet, which prevented T_RM_ differentiation. In addition, their findings showed Runx3 overexpression promotes TILs tumor residency, which led to improved anti-tumor outcomes.

## Epigenetic Remodelling Events During T Cell Exhaustion

The expression of the inhibitory receptor such as PD-1 on T cells suggests that they are vulnerable to respective inhibitory ligands, which are a hallmark of T cell exhaustion (Wherry, 2011). However, exhausted T cell appears to be a functionally heterogeneous population. C-X-C Motif Chemokine Receptor 5 (CXCR5) is a chemokine receptor that is normally present on B cells and CD4^+^ T follicular helper cells (T_FH_) (Im et al., 2016). Tim-3 is recently shown the part of a module that contains multiple co-inhibitory receptors (checkpoint receptors), which are co-expressed and co-regulated on exhausted T cells in chronic viral infections and cancer (Wolf et al., 2020)*.* Im and colleagues have recently shown that CXCR5^+^Tim-3^–^ and CXCR5^–^Tim-3^+^ subsets represent the proliferative and non-proliferative population, respectively, within PD-1^+^ LCMV-specific T cell population (Im et al., 2016). In their study, gene set enrichment analysis (GSEA) showed that CXCR5^–^CD8^+^ T cells were related to CD4^+^ T_H_1 (T helper type 1) cells and CD8^+^ terminal effectors, but the CXCR5^+^ subset was similar to CD4^+^ T_FH_ cells and CD8 memory precursors. Besides, the GSEA result also indicated a relationship between CXCR5^+^CD8^+^ T cells and haematopoietic stem cell progenitors, implying that LCMV-specific CXCR5^+^CD8^+^ T cells may function as memory stem cells during chronic infection. Subsequently, the authors confirmed that CXCR5^+^CD8^+^ T cells were able to proliferate and give rise to the CXCR5^–^Tim-3^+^ subset *in vivo*. However, CXCR5^–^CD8^+^ T cells were terminally differentiated with limited proliferative potential. Specifically, accessibility to *Tcf7* (encoding transcription factor 1, TCF1 protein responsible for the generation of CXCR5^+^CD8^+^ T cells) was reduced, and accessibilities to *Gzmb* (encoding granzyme B) and *Prf1* (encoding perforin-1) were increased during the transition of CXCR5^+^Tim-3^–^ to CXCR5^–^Tim-3^+^ (Jadhav et al., 2019). The high level of perforin and granzyme expression features in CXCR5^–^Tim-3^+^ T cell population suggests their active immune functionality despite poor proliferation (Im et al., 2016). Together, PD-1^+^ virus-specific CD8^+^ T-cell population can be characterized by a unique gene signature with similarities to CD4^+^ T_FH_ cells, CD8^+^ memory precursor cells, and haematopoietic stem cell progenitors. Thus, the epigenetic landscape is closely related to exhausted T cell functionalities.

TILs are shown to experience two significant epigenetic remodeling events rather than one progressive change in chromatin accessibility. Two distinct T cell dysfunction-associated chromatin accessibility states were identified: (1) dysfunctional plastic state and (2) fixed dysfunctional state (Philip et al., 2017). The former appears in the early tumor exposure and is featured by CD38^low^, CD101^low^, CD30L^low^, and CD5^high^ whereas the latter appears in long-term tumor exposure and is featured by CD38^hi^, CD101^hi^, CD30L^hi^, CD5^low^. Despite the fact that PD-1 is expressed in both states, T cells with state (1) retain the responsiveness to interleukin-15 (IL-15), but T cells with state (2) do not. Therefore, whether the exhausted TILs cells can be rescued to be tumor-reactive again, the functional phenotype of the TILs may depend on the epigenetic landscape rather than the quantity of inhibitory ligand surface expression (Philip et al., 2017). Thus far, biological factors in TME that influence the change of chromatin state from (1) to (2) would be interested in the future direction.

H3K79me2 is shown to bind at the Stat5b promoter region and enhance STAT5B transcription that mediates cytokine signallings (such as IL-7 and IL-15) for maintaining CD8^+^ T cell survival (Kelly et al., 2003; Tripathi et al., 2010; Villarino et al., 2017; Bian et al., 2020). Bian et al. comprehensively investigated the abnormal epigenetic patterns correlation with effector T cell malfunction in tumors (Bian et al., 2020). Mechanistically, their results showed that B16F10 cells consumed and outcompeted CD8^+^ T cells for methionine via its high expression of SLC43A2, a methionine transporter, causing the T cells to lose H3K79me2 in a co-culture system. Moreover, their findings showed that the deprivation of methionine promoted CD8^+^ T cell apoptosis and reduced its IFNγ and TNFα production, whereas methionine supplement improved tumor-infiltrating CD8^+^ T cell cytokines production and anti-tumor responses (Bian et al., 2020). This study indicates that methionine consumption is an immune evasion mechanism, and targeting cancer methionine signaling may provide an immunotherapeutic approach (Kelly et al., 2003; Tripathi et al., 2010; Villarino et al., 2017).

One popularly investigated marker transcription factor, TOX, was found to associate with T cell exhaustion. TOX is induced through the Calcineurin-NFAT2 pathway (Macian, 2005; Martinez et al., 2015; Klein-Hessling et al., 2017), but its maintenance is Calcineurin independent in the late-stage (Khan et al., 2019). *Tox* is highly accessible epigenetically in exhausted CD8^+^ T cells compared to naïve, effector, memory phenotypes. Khan et al. showed that knocking out TOX (TOX^−^) in T cells promoted T_E_-associated genes upregulation and downregulated exhaustion associated genes. At the epigenetic level, genetic deletion of TOX resulted in decreased exhaustion-related and memory-related chromatin accessibility and increased T_E_-associated chromatin accessibility.

## Enzymatic Factors Regulating T Cell Function Through Epigenetic Remodeling

It is known that DNA methyltransferase (DNMT) mediates DNA methylation, which implies that DNMT participated in the process of T cell differentiation (Pace and Amigorena, 2020; Akbari et al., 2021). Ladle et al. (2016) have shown that the knocking out DNMT3a gene (DNMT3a^–^) skews early effector CD8^+^ T cells to differentiate into memory precursor phenotype (CD127^+^KLRG1^–^) rather than terminal effector phenotype (CD127^–^KLRG1^+^). It is because DNMT3a binds at the *Tcf7* promoter region to repress the *TCF1* expression, which inhibits the effector CD8^+^ function. It has also been shown that DNMT3a^–^CD8^+^ T cells produce more interleukin-2 (IL-2) and IFNγ in chronic infection settings than those expressing DNMT3a (Ghoneim et al., 2017). Similarly, DNMT1 is critical to T_E_ expansion (Chappell et al., 2006). Knockout of DNMT1 (DNMT1^–^) resulted in significantly fewer IFNγ^+^CD8^+^ T cells than those without knockout upon *in vitro* stimulation (Table 1). However, the cytotoxicity of DNMT1^–^CD8^+^ T cells was enhanced probably because of the demethylation at the perforin enhancer. These findings suggested that the DNA methylome profile of each T cell subset is critical toward T cell functions. The anti-tumor response impacted by intracellular DNMT modulation in CD8^+^ T cells would be an attractive area of research in immunotherapy.

**TABLE 1:** Summary of enzymatic factors and miRNA regulating epigenetics of CD8^+^ T cells with corresponding biological outcomes.

Enzymatic factors	Effect on anti-tumor response	Key biological effect(s)	Reference
DNMT3a	N.A.	DNMT3a (−) skews CD8^+^ T cell differentiation to memory precursors	Ladle et al. (2016)
EZH2	EZH2 positively correlates with anti-tumor responses	EZH2 maintains NOTCH signaling; promotes cytokines (IFNγ, TNFα, GZMB) producing population, and inhibits apoptosis	Zhao et al. (2016)
TET2	TET2 (−) positively correlates with anti-tumor responses	TET2 (−) promotes proliferation and enhance early pro-inflammatory cytokines (IFNγ and TNFα) production in exhaustive (PD-1^+^ and Tim-3^+^) populations	Lee et al. (2021)
*Cbx3*/HP1g	*Cbx3*/HP1g (−) positively correlates with anti-tumor responses	*Cbx3*/HP1γ (−) promotes IFNγ, GZMB, and Perforin 1 expression; *Cbx3*/HP1γ (−) recruited more T_E_ in tumor	Sun et al. (2017)
**miRNAs**			
miR-28	miR-28 positively correlates with anti-tumor responses	miR-28 reduces PD-1^+^ T cell population and recuses its IL-2 and TNF-α production	Li et al. (2016)
miR-150	miR-150 (−) positively correlates with the anti-tumor response	miR-150 (−) skews CD8^+^ T cell differentiation into T_CM_, T_EM_ rather than T_E_; miR-150 (−) enriches cytokines (IFNγ, TNFα, IL-2) producing population	Ban et al. (2017)
miR-491	miR-491 negatively correlated with the anti-tumor response (expected result)	miR-491 reduces CD8^+^ T cell IFNγ production, inhibits proliferation, and promotes apoptosis of CD8^+^ T cells	Yu et al. (2016)
miR-101 and miR-26a	Negatively correlated with the anti-tumor response (expected result)	miR-101 and miR-26a reduce effectors molecules producing population (IFNγ, TNF, GZMB); and promote apoptosis of CD8^+^ T cells	Zhao et al. (2016)
miR-122, miR-149, miR-498, miR-181a/b, miR-3187-3p	Negatively correlated with the anti-tumor response (expected result)	All of each inhibits TNFα secretion by CD8^+^ T cells	Vignard et al. (2020)
miR-92a-3p, miR-21-5p, miR-16-5p, miR-126 and miR-182-5p	Negatively correlated with the anti-tumor response (expected result)	Co-transfection of these miRNA mimics promotes CD8^+^ T cells inhibitory ligands expression (PD-1, CTLA-4, Tim-3, and Lag-3)	Cui et al. (2018)

*N.A., Not applicable; (−) Knock out or inhibition.

Margueron and Reinberg (2011) discussed that polycomb repressive complex 2 (PRC2) silenced gene transcription through H3K27me2 and H3K27me3 modification. H3K27me2 was an intermediate product of H3K27me3 and prevented the acetylation of H3K27. It has been realized that the enhancer of zeste homolog 2 (EZH2), a histone methyltransferase core of PRC2, expresses robustly upon T cell activation (Margueron and Reinberg, 2011; Zhao et al., 2016). Also, EZH2 sustains NOTCH signalings through inhibiting NOTCH suppressor Numb, F-Box, and WD Repeat Domain Containing 7 (FBXW7) through H3K27me3 modification, promoting CD8^+^ T cell polyfunctionality (expressing 2 or 3 effector molecules: IFNγ, TNFα, and GZMB) and suppressing apoptosis (Öberg et al., 2001; Wirtz-Peitz et al., 2008; Yumimoto et al., 2013; Zhao et al., 2016). In terms of anti-tumor immunity, EZH2^+^CD8^+^ T cells are often void of dysfunction markers (KLRG1, Tim-3, CD57), and the EZH2^+^CD8^+^ T cell population positively correlates to anti-tumor response (Wherry, 2011; Crespo et al., 2013; Zhao et al., 2016).

Similarly, ten-eleven translocation 2 (TET2), a methylcytosine dioxygenase involved in DNA demethylation, was found to regulate CD8^+^ T cell functions (Carty et al., 2018; Lee et al., 2021). Upon T cell stimulation, the influx of Ca^2+^ elicits TET2 mRNA expression (Carty et al., 2018). Adoptive transfer of TET2 knocked out (TET2 KO) CD8^+^ T cells to murine melanoma tumor model showed significant tumor regression and a significant increase of TET2 KO CD8^+^ T cell population in the tumor, spleen, and peripheral blood. Functional populations (IFNγ^+^ and TNFα^+^) and exhausted populations (PD-1^+^ and Tim-3^+^) of TET2 KO CD8^+^ T cells were enhanced in the early immune response (3 days) rather than late immune response. Short-term (4 h) *in vitro* cytotoxicity and survival of TET2 KO CD8^+^ T cells were also promoted. At the epigenetic level, the E26 transformation-specific (ETS) transcription factor family was enriched in the differential chromatin-accessible region of TET2 KO CD8^+^ TILs, whereas ETS inhibition reduced the cytotoxicity of TET2 KO CD8^+^ TILs (Lee et al., 2021). These together suggest that the loss of TET2 promotes the accessibility of ETS enriched gene loci which mediate the enhanced anti-tumor function.

H3K9 methylation (e.g., me2 or me3) is an important repressive epigenetic mark on T cell development (Takikita et al., 2016). ESET/SETDB1, one of the major histone methyltransferases, catalyzes H3K9 trimethylation (Schultz et al., 2002; Yang et al., 2002). Takikita et al. (2016) showed that thymocyte-specific deletion of ESET caused impaired T cell development, particularly obvious in CD8 lineage cells that were severely affected. Such deletion led to increased apoptosis and suppressed TCR-induced ERK activation in the CD8^+^ cells. This study outlined the critical role of H3K9 histone methyltransferases in T cell development. Similarly, Sun et al. reported that histone reader *Cbx3*/HP1γ occupied the transcription start site of *Prf1*, *Gzmb*, and *Ifng* in activated CD8^+^ T cells. *Cbx3*/HP1γ insufficiency caused reduced H3K9me3 deposition at these loci, which were subsequently deposited by Runx3 and polymerase II. Adoptive transfer of *Cbx3*/HP1γ insufficient CD8^+^ effector T cells recruited a high population of CD122^+^CD44^+^CD8^+^ effector T cells and NKG2D^+^ CD8^+^ T cells in tumors (Sun et al., 2017). In short, the dynamics of epigenetic profiles actively shape the function and feature of T cells during adaptive immunity. Altering enzymatic factors can skew CD8^+^ T cell effector function. Thus, identifying the mechanism of methylation-associated enzymes remodeling chromatin and DNA methylation in CD8^+^ T cells provides novel insights into therapeutic design T cell-based therapy (Pace and Amigorena, 2020; Akbari et al., 2021; Frias et al., 2021).

## MicroRNA Modulates CD8^+^ T Cell Anti-Tumor Responses

MicroRNA (miRNA) is a non-coding RNA sequence with 18–22 nucleotide units. It is reported that miRNA hybridizes with mRNA to form miRNA/mRNA duplex to restrict mRNA translation and cause mRNA degradation (John et al., 2004; Bushati and Cohen, 2007; Cai et al., 2009; Ha and Kim, 2014). miRNAs are critical to regulating embryonic development and stem cell differentiation. An impaired miRNA profile is often associated with diseases, such as cancers (Yao et al., 2019). Hence, the detection of miRNA expression has recently been an active area of research (Wong et al., 2020; Dai et al., 2021). Moreover, previous reports have shown that miRNAs are differentially expressed in different T cell phenotypes upon T cell activation, implying that miRNA dynamics involves in T cell differentiation. Several excellent reviews have explored the relationship between miRNA dynamics and T cell differentiation (Liang et al., 2015; Emming et al., 2018; Rodríguez-Galán et al., 2018). In this section, we discuss and summarize the latest research progress on how miRNA regulates T cell behaviors (Table 1).

miR-28 targets mRNA of inhibitory receptors PD-1 (Li et al., 2016). Transfecting miR-28 mimetic to exhausted-CD8^+^ T cells reduces PD-1^+^ T cell population and recuses lymphocytes to produce IL-2 and TNF-α. Therefore, miR-28 can be a nucleic acid-based therapeutic approach to shifting T cells from exhausted to non-exhausted phenotype. Also, miR-150 inhibits memory T cell differentiation by targeting 3’ UTR of fork-head box O1 (Foxo1) and therefore suppressing TCF1 (Ban et al., 2017). Ban and colleagues reported that miR-150 deficiency skewed CD8^+^ T cells into T_CM_ and T_EM_ rather than effector T cells (T_E_) phenotype in an acute infection model and enriched the multiple cytokines producing population (e.g., IFNγ, TNFα and IL-2). Moreover, miR-150 deficient memory T cells proliferated more robustly than WT T_M_ cells.

Besides intrinsic miRNA in T cells that cause their exhaustion, tumor cells also secrete biomolecules to suppress anti-tumor activities of T cells and promote their survival. For instance, TGF-β1 secreted by cancer cells upregulates miR-491 in CD8^+^ T cells, subsequently reducing their IFNγ production. miR-491 also limits T cell proliferation by targeting TCF1 and Cyclin-dependent kinase 4 (CDK4) mRNA (Jeannet et al., 2010; Zhou et al., 2010; Lee et al., 2013; Xu et al., 2014; Yu et al., 2016). miR-491 is reported to inhibit B-cell lymphoma-extra large (Bcl-xL) expression, and this downregulation promotes CD8^+^ T cell apoptosis (Yu et al., 2016). Thus, miR-491 is a negative regulator in CD8^+^ T cells for anti-tumor immunity.

miR-101 (Varambally et al., 2008) and miR-26a (Sander et al., 2008) can restrict the aforementioned EZH2 signaling pathway *via* targeting 3’ UTR of EZH2 coding genes and hence suppress NOTCH signalings (Zhao et al., 2016). In TME, the amount of glucose is limited that impaired the glycolysis of TIL, leading to upregulation of miR-101 (Varambally et al., 2008) and miR-26a in TILs (Sander et al., 2008). The transfection of miR-101 or miR-26a mimetic RNA to CD8^+^ T cells reduces stimulating cytokines producing T cell population and promotes T cells apoptosis. Recall that the EZH2^+^CD8^+^ T cell population highly associates with the enhanced anti-tumor response, miR-101 and miR-26a inhibit the expression of EZH2 in CD8^+^ T cells (Zhao et al., 2016). Thus, suppressing both miR-101 and miR-26a in CD8^+^ T cells could be a potential strategy to boost immunotherapy efficiency.

Tumor cells can also deliver miRNA-containing extracellular vesicles (EVs) to inhibit CD8^+^ T cell activities (Ye et al., 2014; Yi et al., 2020). It has been reported that human melanoma cell line-derived EVs contain hsa-miR-122, hsa-miR-149, hsa-miR-498, hsa-miR-181a/b, and hsa-miR-3187-3p. After TILs uptaking the EVs with these miRNAs, their TNFα secretion amount is reduced (Xiao et al., 2012; Vignard et al., 2020). Besides, these EVs can also regulate the expression of miRNA in TILs, which eventually become exhausted. For example, Cui et al. have reported that human leukemia cell line K562-derived EVs contains miR-92a-3p, miR-21-5p, miR-16-5p, miR-126, and miR-182-5p. Co-transfecting with mentioned miRNA mimics to CD8^+^ T cells promotes their inhibitory ligands expression (PD-1, CTLA-4, Tim-3, and Lag-3) (Cui et al., 2018). Hence, inhibiting tumor cells from expressing inhibitory miRNAs that refrain T cell anti-tumor activities can also be one of the future therapeutic strategies in cancer immunotherapy.

## Future Perspective

Traditional clinical trials target single proteins or nodes in biological pathways (e.g., protein inhibitors) but often fail to resolve the diseased phenotypes (Hopkins, 2008). Currently, both miRNA mimics and repressors for therapies are in clinical development or in phase 1–2 clinical trials, although they have not yet been translated into FDA-approved candidates (Hanna et al., 2019). Nevertheless, both traditional and miRNA therapeutics provoke off-target biological effects by the pleiotropic nature. Similarly, enzymatic factor-based therapeutics, such as DNMT inhibitors, have been recognized as novel strategies for cancer therapy via epimutations (on cancer cells or Tregs), yet they are still in the developmental stage (Hu et al., 2021). Intriguingly, highly proliferative cells, including cancer cells, are shown to be very sensitive to hypomethylating agents, i.e., DNMT inhibitors (e.g., azacytidine, decitabine, and zebularine), which also potentiate the effects of radiotherapy for cancers by exposing nucleoside analogues as radiosensitizers (Gravina et al., 2010). These findings imply that these inhibitors are on-target and can overcome melanoma resistance to immunotherapy (e.g., PD-1/PD-L1 interactions). However, the report of the effect of these inhibitors on T cells has been limited despite the intensive studies of overexpressing/knocking out the related genes responsible for DNA methylation/demethylation via biological means. Also, exhausted T cells are much less proliferative, suggesting a low targeting effect by DNMT inhibitors in the TME (Gravina et al., 2010). In addition, literature indicated that DNMT inhibitors caused toxicity and possibly provoked mutagenic/carcinogenic potentials to non-cancerous cells in the long term (Eden et al., 2003). Together, selective delivery of enzymatic drugs or miRNAs is crucial to limit off-target effects and optimize the outcome of cancer immunotherapy.

To facilitate on-target delivery of miRNA for reversing T cell exhaustion, we believe that cytocompatible nanomaterials such as liposomes and synthetic polymers are effective miRNA vehicles, and their surfaces can be modified with T cell-targeting ligands (e.g., CD8 binding ligands) to increase intracellular controlled released, thereby enhancing the therapeutic efficacy (Li et al., 2019; Hou et al., 2021). However, studies employing nanomaterials for intracellular delivery of miRNA in T cells have been rare to increase tumor response rates to immunotherapy. Similarly, these nanocarriers are also suitable to encapsulate proteins for controlled releasing the enzymatic factors/inhibitors. For instance, a recent study fabricated FDA-approved polymers poly(lactic-co-glycolic acid) (PLGA) and polyethylene glycol (PEG) hybrid nanoparticles bearing CD8a and PD-1 antibodies to target CD8^+^/PD-1^+^ T cells for specifically delivering TGFβ (an immunosuppressor) inhibitors extending the survival and sensitivity to PD-1 antibodies in TILs (Schmid et al., 2017). Thus, we suggest that the recent advances in nanotechnology provide a highly sound footing platform to foster the integration of epigenetic regulation in cancer immunotherapy.

## Conclusion

CD8^+^ T cells recognize and can potentially eradicate cancer cells in adaptive immunity. Upon T cell activation, phenotypical and functional change dramatically and progressively. T cell differentiation is accompanied by changes in the epigenetic profile. Enzymes and miRNAs are the two discussed factors that control the change in T cell phenotype and function acquisition in this review. Besides, the interactions between TILs and tumor cells in TME can cause sophisticated functional changes in TILs through epigenetic remodeling. Therefore, understanding the mechanisms of T cell dysfunction at the epigenetic level can potentially explore the conceptual gaps in knowledge for the failure of conventional T cell-based cancer immunotherapy. With the recent advances in nanotechnology, the efficacy of such therapy can potentially be optimized.

## Glossary

**Bcl-xL**: B-cell lymphoma-extra large
***Cbx3*/HP1γ**: Chromobox homolog 3/heterochromatin protein 1 gamma
**CCR7**: CC-chemokine receptor 7
**CDK4**: Cyclin-dependent kinase 4
**CTLA-4**: Cytotoxic T-lymphocyte-associated antigen 4
**CXCR5**: C-X-C Motif Chemokine Receptor 5
**DCs**: Dendritic cells
**DNMT**: DNA methyltransferase
**ETS**: E26 transformation-specific
**EVs**: Extracellular vesicles
**EZH2**: Enhancer of zeste homolog 2
**FBXW7**: F-Box and WD Repeat Domain Containing 7
**Foxo1**: Fork-head box O1
**GZMB**: Granzyme B (encoded by *Gzmb*)
**H3K4me3**: Trimethylation of lysine 4 on histone H3
**H3K9ac**: Acetylation of lysine 9 on histone H3
**H3K9me2**: Dimethylation of lysine 9 on histone H3
**H3K9me3**: Trimethylation of lysine 9 on histone H3
**H3K27me2**: Dimethylation of lysine 27 on histone H3
**H3K27me3**: Trimethylation of lysine 27 on histone H3
**H3K79me2**: Dimethylation of lysine 79 on histone H3
**IFNγ**: Interferon-gamma (encoded by *Ifng*)
**IL-2**: Interleukin-2 (encoded by *IL2*)
**IL-15**: Interleukin-15
**LAG-3**: Lymphocyte-activation gene 3
**LCMV**: Lymphocytic choriomeningitis virus
**NFAT**: Nuclear factor of activated T-cells (NFAT2 is encoded by *Nfatc1*)
**NKG2D**: Natural killer group 2 member D
**KLRG1**: Killer Cell Lectin Like Receptor G1
**PD-1**: Programmed Cell Death Protein 1
**PD-L1**: Programmed death-ligand 1
**pMHC**: Peptide-major histocompatibility complex
**PRC2**: Polycomb repressive complex 2
**Runx3**: Runt-related transcription factor 3T_M_ cells: Memory T cells
**T_N_ cells**: Naïve T cells
**T_SCM_ cells**: Memory stem T cells
**T_CM_ cells**: Central memory T cells
**T_EM_ cells**: Effector memory T cells
**T_E_ cells**: Effector T cells
***Tbx21***: T-box transcription factor 21 coding gene
**TCF1**: T cell factor 1 (encoded by *Tcf7*)
**TCR**: T cell receptor
**TET2**: Ten-eleven translocation-2
**TGFβ**: Transforming growth factor beta
**TILs**: Tumor infiltrating lymphocytes
**Tim-3**: T cell immunoglobulin and mucin domain-containing protein-3
**TME**: Tumor microenvironment
**TNFα**: Tumor necrosis factor-alpha
**TOX**: Thymocyte Selection Associated High Mobility Group Box (encoded by *Tox*)
